# Cross-sectoral primary care-based approaches to reducing suicides in England

**DOI:** 10.1177/01410768231220306

**Published:** 2024-01-04

**Authors:** Faraz Mughal, Aziz Sheikh

**Affiliations:** 1 School of Medicine, Keele University, Keele, ST5 5BG, UK; 2 Usher Institute, University of Edinburgh, Edinburgh, EH8 9AG, UK

Recent data indicate that there were 5275 deaths by suicide in England in 2022 (at a rate of 10.6 per 100,000 (95% CI: 10.3–10.9)) with no evidence of declines since 2020, a year after the publication of the 2019 Long Term Plan, which made suicide prevention a priority for the National Health Service (NHS).^1,2^ This has led to the Department of Health and Social Care publishing a new cross-sectoral strategy for suicide prevention for England in September 2023.^3^

This new strategy aims to reduce the rates of suicide in England over the next five years, targeting an initial reduction within half this time.^3^ It was informed by discussions with key stakeholders and seeks to reduce suicide rates by supporting investments through the NHS, voluntary sector and community sector in population-level interventions addressing social isolation and loneliness, targeting support to high-risk groups, reducing access to means of suicide and the provisioning of real-time clinical and social data to support frontline decision making for at-risk patients. The strategy is much broader than those previously formulated as evidenced by, for example, plans to support employers in prioritising employee wellbeing, unlocking investments from the private and third sectors, and improving training across the justice system.

Primary care is highlighted as a setting where people at risk of suicide need to be better supported, echoing calls from the World Health Organization, which has emphasised the vital role primary care has in reducing rates of suicide.^4^

But how can primary care help lower suicide rates? The National Confidential Inquiry into Suicide and Safety in Mental Health identified that suicides recorded in primary care records were associated with high consultation rates and undiagnosed mental illness.^5^ A study of linked electronic healthcare records (EHR) across primary and secondary care in Wales found that in 71% of deaths by suicide the last healthcare contact was in general practice.^6^ Of middle-aged men who died by suicide, 43% had a general practitioner consultation in the three months before suicide, and over half presented with a mental health problem.^7^ Rates of self-harm coded in UK general practice EHR have been increasing over time, in particular for young people, and especially teenage girls in early coronavirus disease 2019.^8,9^ This evidence indicates an opportunity to identify high-risk individuals in the primary care setting early, and target personalised support to unmet need.

We do acknowledge that there are current challenges in primary care that may make this difficult to achieve but we see three key opportunities that need to be availed. First, at the practitioner level, clinicians should utilise the ‘every contact counts’ approach for patients presenting after self-harm and in suicidal distress, especially in high-risk groups, such as young people and new mothers, and tailor treatments to clinical need. Clinicians should also be alert to prescribed medications, such as tricyclic antidepressants, opiates and benzodiazepines, in patients at risk of suicide, and review their indication to reduce the risk of future (fatal and non-fatal) self-poisoning. Second, there is the need for the creation of integrated primary care systems where a culture of patient safety, kindness and compassion is at its core. Primary care teams, serving their region’s needs, are diversifying in staff type and delivery of care and while this supports rising patient demand, continuous education is crucial to foster a culture of caring that prioritises the safety of all patients.^10^ Third, primary care requires national investment to support public mental health approaches, develop new research infrastructure, such as data linkage, with mental health services and prisons and test new models of care for patients in suicidal crisis involving supporting services. Such a model of care could involve clinicians referring patients in crisis to a specialist mental health nurse for prompt assessment and management within primary care – an approach that has shown promising initial evaluation in Ireland.^11^

In England, unfortunately, each year people continue to die by suicide with devastating consequences for their families, friends and clinicians. With its longitudinal, person- and family-centred approach to care provision, primary care needs to be at the heart of efforts to reduce the currently unacceptable high rates of suicide. The new *Suicide prevention in England* strategy offers an opportunity to leverage the considerable expertise and assets in primary care, through cross-sectoral approaches, to reduce the numbers of people losing their lives to the tragic but preventable tragedy of suicide.

## Declarations

### Guarantor

FM.

### Provenance

Not commissioned; editorial review.
